# Supplementary material for: Identification of photoperiod-induced specific miRNAs in the adrenal glands of Sunite sheep (Ovis aries)
Source: Front Vet Sci. 2022 Jul 22;9:888207. doi: 10.3389/fvets.2022.888207 (PMC9354845; doi:10.3389/fvets.2022.888207)
Supplement: Supplementary file 1 [file Data_Sheet_1.ZIP › Supplementary file/miRDeep2 file.zip!/miRDeep2 file/Novel_Structure/SP42A2/Novel_3.pdf]

Provisional ID	:	NC_019476.2_34385
Score total	:	0.6
Score for star read(s)	:	-1.3
Score for read counts	:	-1.3
Score for mfe	:	1.7
Score for randfold	:	1.6
Score for cons. seed	:	
Total read count	:	9
Mature read count	:	8
Loop read count	:	0
Star read count	:	1

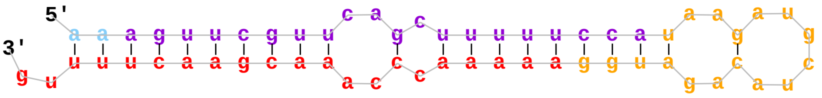

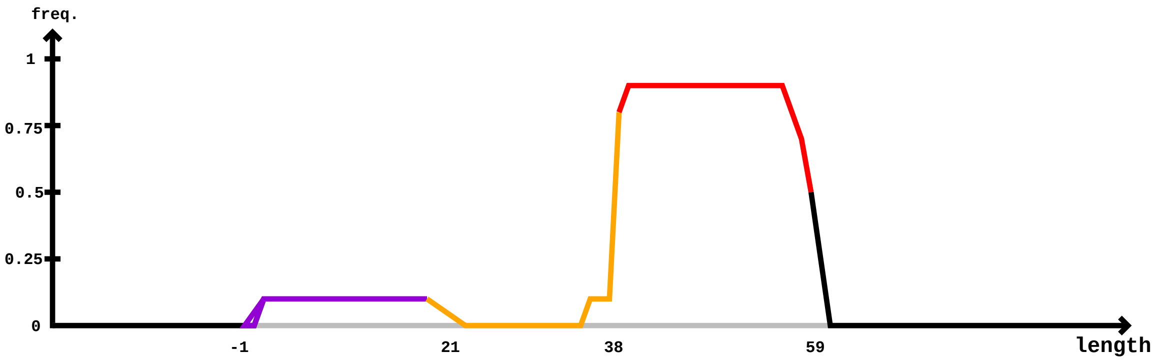

Star Mature

```
5'- ucauacuggauuggccgaaaaguucguucagcuuuuuccauaagaugcuacagauggaaaaacccaaacgaacuuuugggccaaccuaauacaaagacuuguggaccugc   -3'    obs
    ucauacuggauuggccgaaaaguucguucagcuuuuuccauaagaugcuacagauggaaaaacccaaacgaacuuuugggccaaccuaauacaaagacuuguggaccugc          exp
    .......((.((((((.(((((((((((..(.(((((((((..(......)..))))))))).)..))))))))))).))))))))...(((((....))))).......   reads      mm        sample
    ....................aguucguucagcuuuuucca......................................................................   1          0         seq
    ......................................................uggaaaaacccaaacgaacuu...................................   1          0         seq
    .........................................................aaaaacccaaaUgaacuu...................................   1          1         seq
    .........................................................aaaaacccaaaUgaacuuuu.................................   1          1         seq
    .........................................................aaaaacUcaaacgaacuuuug................................   5          1         seq
    ..........................................................aaaaUccaaacgaacuuuu.................................   1          1         seq
```
